# Prevalence, molecular characterization of *Staphylococcus aureus* isolated from cheese and in vitro antibacterial activity of silver nanoparticles against such strains

**DOI:** 10.14202/vetworld.2015.908-912

**Published:** 2015-07-26

**Authors:** Karima G. Abdel Hameed, Mona A. El-Zamkan

**Affiliations:** Department of Food Hygiene and Control, Faculty of Veterinary Medicine, South Valley University, Qena, Egypt

**Keywords:** antibacterial activity, multiplex polymerase chain reaction, *Staphylococcus aureus*, silver nanoparticles

## Abstract

**Aim:**

The aim was to investigate cheese samples for the prevalence of *Staphylococcus aureus*, evaluate multiplex polymerase chain reaction (PCR) methods for *S. aureus* identification, as well as to determine the antibacterial activity of silver nanoparticles against such strains.

**Materials and Methods:**

Total of 100 random locally manufactured cheese samples were collected from Qena dairy markets, Egypt, and examined conventionally for the prevalence of *S. aureus* then, confirmation of these isolates were done using multiplex PCR. The antibacterial activity of silver nanoparticles against such isolates was also checked.

**Results:**

Lower prevalence of *S. aureus* in Damietta cheese (54%) than in Kareish cheese (62%) was recorded. As well lower frequency distribution for both *S. aureus* (36%) and CNS (8%) was also reported for Damietta cheese. Using of multiplex PCR method for *S. aureus* identification have been confirmed all 58 *S. aureus* stains that were identified conventionally by detection of two PCR products on agarose gel: The 791 bp and the 638 bp. The correlation coefficient between conventional and multiplex PCR method was 0.91 and was significant at p≤0.001. Regarding antibacterial activity of silver nanoparticles using disk diffusion method on Baird Parker agar it was found that inhibition zone of silver nanoparticles against *S. aureus*, was 19.2±0.91 mm and it was higher than that produced by gentamicin (400 units/ml) 15.2±0.89 mm.

**Conclusions:**

The present study illustrated the higher prevalence of *S. aureus* in cheese samples that may constitute a public health hazard to consumers. According to the results, it can be concluded that silver nanoparticles can be used as an effective antibacterial against *S. aureus*. Thereby, there is a need for an appropriate study for using silver nanoparticles in cleaning and disinfection of equipment and in food packaging.

## Introduction

Damietta cheese is the most popular type of pickled soft cheese by all socioeconomic classes in Egypt due to its nutritional value, convenience and good taste. Fully ripened Damietta cheese has strong, sharp flavor, as well as smooth body and texture [1]. Furthermore, Kareish cheese is considered as one of the most popular local types of fresh soft cheese in Egypt. The increasing demand for it by Egyptian consumers mainly attributed to its high protein content and low price. The traditional method for cheese production affords many opportunities for microbial contamination. It is generally made from raw buffaloes’ or cows’ milk which is often of poor microbiological quality owing to the high microbial load present in raw milk and the unsatisfactory conditions under which it is produced. Therefore, it can be considered as a good medium for the growth of different types of spoilage and pathogenic microorganisms [2].

*Staphylococcus aureus* (*S. aureus*) is a leading cause of gastroenteritis resulting from the consumption of contaminated food [3]. The accurate assessment of *S. aureus* depends on the phenotypic characterization of cultured bacteria. Recently, molecular methods allow more information regarding contamination and dissemination of *S. aureus* in the food chain. Most PCR protocols focused on amplification of conserved regions of eubacterial rRNA genes. Other protocols were focused on amplification of genes found only in this species. Hence, multiplex PCR is a very efficient method for confirming phenotypic identification results of *S. aureus* in cultures [4].

Resistance of *S. aureus* to antimicrobial agents is a well-documented in dairy cows. Because of resistance to antimicrobial agents, there is a growing interest in using an alternative antimicrobial agent.

Silver nanoparticles have received special attention as a possible antimicrobial agent [5]. As well as, it can potentially be used in antimicrobial packaging applications to preserve the quality of food products [6].

Therefore, the aim of the present study was to allow qualitative checking of hygienic conditions of the examined cheese samples for the prevalence of *S. aureus* in Qena Governorate, evaluate multiplex PCR methods for *S. aureus* identification, as well as to assess the antibacterial activity of silver nanoparticles against cheese derived *S. aureus*.

## Materials and Methods

### Ethical approval

Ethical approval is not required to pursue this type of study.

### Design of study

The present study was carried out within (January - October) 2014 in the Department of Food Hygiene, Faculty of Veterinary Medicine, South Valley University, Qena, Egypt.

### Samples collection

A total of 100 random locally manufactured cheese samples were collected from Qena dairy markets, Egypt including Damietta, and Kareish cheese (50 samples each) under aseptic conditions. The samples were collected in dry, clean, and sterile glass containers, and sent immediately in an ice box to the laboratory.

### Samples preparation

A total of 10 g of each cheese sample (125 g) were homogenized in 90 ml of 0.1% peptone water using a stomacher (A. J. Seward, London, UK) at 45°C for 2 min.

### Isolation and identification of *S. aureus*

A volume of 1 ml of the homogenate was inoculated into a sterile test tube containing 10 ml of brain heart infusion broth (BHI) (Bio-Merieux) and was incubated at 37°C for 48 h. 0.1 ml of the incubated broth was streaked into plates of selective media Baird - Parker agar [7] and incubated at 37°C for 2 days (Oxoid). The suspected colonies were inoculated into the slope of the nutrient agar for morphological and biochemical tests. The identification was carried out using the following tests: Gram staining, production of coagulase, catalase, and fermentation of mannitol [8].

### DNA extraction and multiplex PCR amplification

DNA extraction and PCR amplification were done [9]. Typical *S. aureus* (5-8) colonies were selected purified and were subject for DNA extraction following grown up in the BHI at 37°C for 24 h. One ml of incubated broth was centrifuged at 8000 g for 7 min. The NucleoSpin^®^ Tissue Kit was used to obtain bacterial DNA, according to the manufacturer recommendations. The bacterial DNA was next frozen until the use for the multiplex PCR. The DNA amplification was performed using primers given by William *et al*. [4].

The primers sequences were as follows:

**Table:** 

Target gene	Primers	Length (bp)
Staph A	5’CCTATAAGACTGGGATAACTTCGGG3’	791 bp
Staph B	5’CTTTGAGTTTCAACCTTGCGGTCG3’	
*Clf*A	5’GCAAAATCCAGCACAACAGGAAACGA3’	638 bp
*Clf*B	5’CTTGATCTCCAGCCATAATTGGTGG 3’	

The PCR reaction was conducted in a GeneAmp PCR System 9600 Thermal Cycler (AB). The cycling parameters were: (1) 94°C for 10 min, (2) 94°C for 1.5 min, (3) 55°C for 1 min, (4) 72°C for 1 min, 36 cycles of step 2 through 4 inclusive, and 72°C for 10 min. The PCR reaction was done using a mix containing: 50 - 100 ng of genomic DNA, 200 µM of each dNTP, 3.0 mM MgCl_2_, 20 pmol of each primer, 2.5 U Taq Gold polymerase (AB). The PCR products were visualized using a 2.5% agarose gel 20 containing 0.5 µg of ethidium bromide per ml in relation to the DNA mass ladder standard (DNA from pUC19, 11-1444 bp, BTL).

### Synthesis of silver nanoparticles

Silver nanoparticles that were synthesized as follow, (4 ml cow’s milk was mixed with 96 ml of 1 mM silver nitrate solution then the mixture was incubated for 8 h using rotatory shaker (180 rpm) at room temperature) and biologically characterized [10], were kindly provided from the Department of Chemistry Faculty of Sciences Assiut University.

### Antibacterial assay

A total of 59 *S. aureus* strain that were isolated from cheese samples surveyed in this study and were confirmed by multiplex PCR were used to assess the antibacterial activity of silver nanoparticles by the disc diffusion method on Baird Parker agar. The organisms were propagated in BHI and incubated at 37°C for 24 h then 0.1 ml of the inoculated broth was streaked into the plat. 50 μl of freshly prepared silver nanoparticles were added onto a filter paper of 5 mm diameter. The filter paper was then put into the inoculated plates. Control plates free of silver nanoparticles were used. The plates were left at 4°C for 15 min to allow diffusion then incubated at 37°C for 24 h. The inhibition growth zone diameter was measured in mm with a ruler. The antibacterial activity was evaluated according to the following criteria: Zone of inhibition range >18 mm showed significant activity, 16-18 mm good activity, 13-15 mm low activity, 9-12 mm non-significant activity, and <8 mm no activity [11]. The cultures that were inhibited by the silver nanoparticles were also subjected to the antimicrobial activity experiment with gentamicin (400 units/ml) as standard antibacterial drug [12].

### Statistical analysis

The prevalence of *Staphylococcus* species was calculated by dividing the number of positive samples by the total number of examined samples. Data were entered into the Microsoft Excel Spreadsheet. Comparison between rapid PCR and conventional methods for species identification was done by Pearson’s correlation coefficient (SAS program). Antibacterial activity results represented as means ± standard error and were analyzed using Analysis of Variance.

## Results and Discussion

Most of locally manufactured dairy products are liable to be contaminated with different types of microorganisms from different sources include milking, manufacture, storage, transportation, and distribution. Therefore, such products may constitute a public health hazards.

In the present study, 58 *S. aureus* strains were isolated and identified from both types of the examined cheese samples (Table-1). According to the results, it was found that the higher prevalence of *S. aureus* in Kareish than in Damietta cheese. This could be due raw milk used in the manufacture of Kareish cheese and the primitive way of processing, handling and methods of selling. On the contrary, salt and ripening in Damietta cheese, cause microbial cell dehydration and interfere with the action of proteolytic enzyme [13].

**Table-1:** Prevalence of *S. aureus* and *CNS in the examined cheese samples.

Cheese samples	Number of examined samples	N (%)		

		Positive samples	Isolated strains	

			*S. aureus*	*CNS
Damietta	50	33 (66)	27 (54)	6 (12)
Kareish	50	42 (84)	31 (62)	11 (22)
Total	100	75 (75)	58 (58)	11 (11)

* CNS=Coagulase negative staphylococci, *S. aureus=Staphylococcus aureus*

Table-1 shows that the prevalence of *S. aureus* in Damietta cheese was 54%. Higher results of (70%) and (58%) were obtained by Mohammed; Saad [2,14], respectively. Concerning the prevalence of *S. aureus* in Kareish cheese it was 62% (Table-1). Higher results of (94%) and (93%) were recorded by Mohammed [2] and Saad [14], respectively.

The presence of *S. aureus* in food would not be enough to estimate risk because viable cells can be inactivated by heat treatment. However, with those types of cheese that made by raw milk as in (kareish cheese) or by improper heat treated milk as in (Damitta cheese) the risk of contamination is more serious and the possibility to perform toxins is more expected.

Therefore, in this study, we discussed *S. aureus* as an important pathogen that promote cellular attachment, invasion as a virulence factors, and antibiotic resistance point of view.

Table-2 shows that the lower frequency distribution of both *S. aureus* and CNS was reported for Damietta cheese. The presence of *S. aureus* in cheese samples might be attributed to inadequate heat treatment, unhygienic handling practices, use of dirty containers, faulty storage, and transportation [15]. Low prevalence of CNS was detected in the examined samples. CNS possesses less virulence factors than *S. aureus*. Moreover, there is no evidence in the literature discussing that CNS isolated from milk or dairy products has ever been involved in a case of food poisoning or human pathology following ingestion of dairy products.

**Table-2:** Frequency distribution of *S. aureus* and CNS in the examined cheese samples.

Cheese samples	Isolated strains		*S. aureus*		CNS	
		
	No./75	%	No./75	%	No./75	%
Damietta	33	44	27	36	6	8
Kareish	42	56	31	41.3	11	14.6

CNS=Coagulase negative staphylococci, *S. aureus=Staphylococcus aureus*

Regarding PCR method for *S. aureus* identification, two genes have been analyzed. The first, corresponding to regions of the 16S rRNA genes that is conserved among staphylococci and unique by comparison to the other eubacterial species. The second corresponds to the *S. aureus* clfA virulence gene, which encodes a surface-exposed fibrinogen-binding protein [16]. The choice of clfA virulence gene was based on the fact that clfA is present only in the genome of all *S. aureus* strains [17]. Gene size was determined in base pairs (bp) comparing the length of the PCR product.

**Figure-1:**
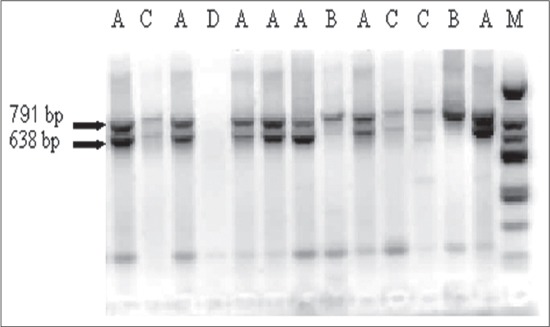
Specificity of multiplex polymerase chain reaction, Line M - molecular size marker (11-1444 bp), Line A - sample containing *Staphylococcus aureus* strain (791 and 638 bp). Line B - sample containing other *Staphylococcus* species (638 bp), Line C - other bacterial species, Line D - control sample (contain no DNA).

Molecular characterization revealed that all the recovered isolates were positive for clfA virulence gene that amplified at 638 pb (Figure-1). This is consistent with the fact that this gene is ubiquitously carried by different *S. aureus* and plays a determinant role in bacterial virulence [18].

As shown in Table-3, conventional methods identified 58 bacterial isolates as *S. aureus*, all of them were confirmed by PCR by detection of two PCR products on agarose gel: the 791 bp and the 638 bp (Figure-1 - Line A). Among the 17 CNS isolates identified by conventional methods 13 of them were confirmed by PCR, and indicated on the gel by detection of one PCR product (791 bp) (Figure-1 - Line B). Only 4 of CNS isolates was not identified correctly by PCR and these strains were classified as follow, one was classified as *S. aureus* and 3 isolates as infection by other bacterial species because this isolate failed to yield any amplification product on the gel (Figure-1 - Line C). The possible explanation of this is the tube coagulase test used as conventional method to identify *S. aureus* in this study is based on coagulation of rabbit plasma after 2-4 h of incubation and an overnight incubation is necessary to obtain reliable results. The long incubation time may allow coagulation of plasma and then disappear rapidly without accurate observation; however, it remains possible that this isolate is tube coagulase negative where, some tube coagulase negative *S. aureus* have been reported by Kelvin *et al*. [19]. These observations suggest that the discrepancy was probably due to a negative coagulase test rather than a PCR failure.

**Table-3:** Bacterial isolates as diagnosed by conventional and by PCR methods.

Diagnosis by conventional methods		Diagnosis by PCR method	
	
Bacterial strains	No.	Confirmed•	Not confirmed••
*S. aureus*	58	58	0
CNS	17	13	4
Total	75	71	4

• Number of bacterial isolates identified by conventional method and confirmed by PCR method,

•• Number of bacterial isolates identified by conventional method but not confirmed and had another classification by PCR method, PCR=polymerase chain reaction, *S. aureus=Staphylococcus aureus*

The correlation coefficient between conventional and PCR method was 0.91 and was significant at p≤0.001. Similar specificity of the multiplex PCR protocol was obtained by William *et al*. [4] and Hameid [9].

PCR method for identification of *S. aureus* seems to be objective, sensitive, and specific when compared to conventional methods. Furthermore, it can be used not only for unambiguous identification of *S. aureus* but also for the identification of other *Staphylococcal* species as it discriminate between closely related species.

Antimicrobial resistant of *S. aureus* can be transmitted by different foods, including contaminated milk through the ingestion of antibiotic residues in food [20]. Recently, silver nanoparticles is one of the non-toxic and safe antibacterial agents to the body and have been investigated as very strong bactericidal activity against gram-positive as well as gram-negative bacteria including multiresistant strains, as well as potential antifungal agent [21].

Table-4 shows the inhibition zone of *S. aureus* growth caused by silver nanoparticles as compared with those caused by gentamicin. Furthermore, gentamicin (10 µg) was previously evaluated for its effect against *S. aureus* by Hameed and El-malt [22] and the susceptibility of 90.32% was obtained. Silver nanoparticles were more effective against *S. aureus*, with zones of inhibition of 19.2 ± 0.91mm than gentamicin (400 units/ml) with zones of inhibition of 15.2±0.89 (Table-4).

**Table-4:** Zone inhibition (mm) of *S. aureus* isolates growth by gentamicin and by silver nanoparticles.

Type of antibacterial	Minimum	Maximum	Mean±SE
Gentamicin (400 units/ml)	13	17	15.2±0.89
Silver nanoparticles	17.9	21.3	19.2±0.91

*S. aureus=Staphylococcus aureus*, SE: Standard error

We used gentamicin as a standard antibacterial against *S. aureus* [12] as well as according to our previous study by Hameed and El-malt [22] we found that among 12 tested antibiotics, gentamycin was the most effect against *S. aureus* isolated from raw milk and ice cream.

It is clear that silver nanoparticles displayed antimicrobial activity toward the tested *S. aureus* strains. Similarly, Dehkordi *et al*. [23] and Kazemi *et al*. [24] recorded the antibacterial activity of silver nanoparticles against *S. aureus*. The ability of silver nanoparticles to inhibit the growth of *S. aureus* may be due to the release of antibacterial ions from the nanoparticles surfaces as reviewed by Seiln and Webster [25]. The silver ions with positive electricity are able to bind to bacteria cell wall, thereby its damage.

According to the result obtained in this study and from literature review [26,27], it can be concluded that silver nanoparticles can be used as antibacterial against *S. aureus* as well it could be a good alternative for cleaning and disinfection of equipment and surfaces in food-related environments as well as in silver-incorporated food packaging.

## Conclusion

Cheese samples obtained from Qena city markets constitute a high-risk hazard to consumers due to the high prevalence of *S. aureus*. Therefore, strict hygienic measures during manufacture, storage, and distribution of the cheese should be applied to ensure high-quality dairy products. PCR method for confirmation of *S. aureus* was objective and sensitive. Silver nanoparticles have an antibacterial effect against the tested *S. aureus*.

## Authors’ Contributions

KGAH conceived, designed the study and drafted the manuscript. KGAH and MAE collected and analyzed samples. Both authors read and approved the final manuscript.
